# A Novel Dielectric Modulated Gate-Stack Double-Gate Metal-Oxide-Semiconductor Field-Effect Transistor-Based Sensor for Detecting Biomolecules

**DOI:** 10.3390/s23062953

**Published:** 2023-03-08

**Authors:** Dibyendu Chowdhury, Bishnu Prasad De, Bhargav Appasani, Navaneet Kumar Singh, Rajib Kar, Durbadal Mandal, Nicu Bizon, Phatiphat Thounthong

**Affiliations:** 1 Department of ECE, Haldia Institute of Technology, Haldia 721657, India; 2 School of Electronics Engineering, KIIT University, Bhubaneswar 751024, India; 3 University College of Engineering and Technology (UCET), Vinoba Bhave University (VBU), Hazaribag 825301, India; 4 Department of ECE, NIT Durgapur, Durgapur 713209, India; 5 Faculty of Electronics, Communication and Computers, University of Pitesti, 110040 Pitesti, Romania; 6 Doctoral School, University Politehnica of Bucharest, 060042 Bucharest, Romania; 7 ICSI Energy, National Research and Development Institute for Cryogenic and Isotopic Technologies, 240050 Ramnicu Valcea, Romania; 8 Renewable Energy Research Centre (RERC), Department of Teacher Training in Electrical Engineering, Faculty of Technical Education, King Mongkut’s University of Technology North Bangkok, Bangkok 10800, Thailand; 9 Group of Research in Electrical Engineering of Nancy (GREEN), University of Lorraine-GREEN, 54000 Nancy, France

**Keywords:** dielectric modulation, threshold voltage, sensitivity, biomolecules, DG MOSFET, GSDG MOSFET, biosensor

## Abstract

In this article, the performance of n-type junctionless (JL) double-gate (DG) MOSFET-based biosensors with and without gate stack (GS) has been studied. Here, the dielectric modulation (DM) method is applied to detect biomolecules in the cavity. The sensitivity of n-type JL-DM-DG-MOSFET and n-type JL-DM-GSDG-MOSFET-based biosensors have also been evaluated. The sensitivity (ΔV_th_) improved in JL-DM-GSDG MOSFET/JL-DM-DG-MOSFET-based biosensors for neutral/charged biomolecules is 116.66%/66.66% and 1165.78%/978.94%, respectively, compared with the previously reported results. The electrical detection of biomolecules is validated using the ATLAS device simulator. The noise and analog/RF parameters are compared between both biosensors. A lower threshold voltage is observed in the GSDG-MOSFET-based biosensor. The I_on_/I_off_ ratio is higher for DG-MOSFET-based biosensors. The proposed GSDG-MOSFET-based biosensor demonstrates higher sensitivity than the DG-MOSFET-based biosensor. The GSDG-MOSFET-based biosensor is suitable for low-power, high-speed, and high sensitivity applications.

## 1. Introduction

The DG MOSFET-based biosensors with DM techniques are used for label-free sensing of biomolecules (neutral and charged) by making a cavity at the gate region. Sharma et al. [1] investigated the electrical performances of different types of DG MOSFET by applying channel engineering and gate-stack engineering. GCGSDG is helpful for high-speed switching applications. GSDMDG shows excellent performance as an amplifier. The electrical behaviours of DG MOSFET [2] are used for RF and analog applications. The analytical model for the multiple-gate MOSFETs was proposed in [3]. GAA MOSFET performs superior to DG MOSFET regarding threshold voltage and DIBL effect. Pati et al. [4] reported the RF performance of the underlap double-gate MOSFETs taking the variation of body and oxide thickness. The process-dependent parameters (PDPs) have significant effect on the analog and RF performance of underlap double-gate MOSFET (UDG-MOSFET). The electrical performance of DG MOSFETs [5] was optimized using the evolutionary technique MOGA. It is observed that the proposed MOGA-based approach provides promising results. Swain et al. [6] explored the RF performance of GCGS DG MOSFETs by optimizing the channel length and thickness of the high-k oxide. Proper optimization of those parameters has a significant role in low-power applications. The electrical behaviours of GS DG MOSFET using evolutionary techniques, such as CRPSO and ALC-PSO, are reported in [7]. Both CRPSO and ALCPSO have efficiently found the optimal dimensions and enhanced the electrical performance parameters for GSDG MOSFET. Ghosh et al. [8] studied cylindrical-gate MOSFET’s linearity and intermodulation distortion. Gate-material-engineered cylindrical-gate MOSFET (GME CGT MOSFET) provides high linearity. In [9], a sensing metric is proposed for a FET-based biosensor to decrease data fluctuations. This sensing metric has shown improved SNR and reduced process variations. Kim et al. [10] proposed an analytical method to describe the electrical properties of biomolecules for an asymmetric DG FET. The proposed technique was capable of extracting the permittivity and charge density of biomolecules electrically. In [11], the performance of DG MOSFET-based biosensors was studied using dielectric modulation. Verma et al. [12] investigated the performance of the vertical dielectrically modulated tunnel FET-based biosensor. In [13], an analytical model was reported to expose the biological molecules with the help of a dielectric modulation technique for both the n or p channel JL DM DG MOSFET-based biosensor. It has been reported that for the negatively (positively) charged biomolecules, n-type (p-type) JL-DM-DG-MOSFET shows better sensitivity. Mendiratta et al. [14] studied the sensing performance of an n^+^ pocket asymmetrical junctionless DG-MOSFET-based biosensor. The proposed biosensor can be used for detecting diseases. In [15], the effect of the gate material at the cavity was studied for the biosensor based on dual-material DG-JL-MOSFET. The simulation results proved that the optimization of gate metal work functions enhances the sensitivity of the biosensor. The sensitivity of different DG-MOSFET-based biosensors is discussed in [16,17]. Different FET-based biosensors are reported in [18,19,20,21,22,23,24]. Makarona et al. [25] fabricated metal-insulator-semiconductor (MIS) devices, including self-assembled molecular monolayers (SAM). A dielectric-modulated FET [26] is used for biosensing. Jang et al. [27] presented a vertical gold nanogap to detect protein–ligand binding. The electrical parameters are evaluated for metal–pentacene–insulator–semiconductor structures in [28]. Chandra et al. [29] reported the fabrication technique for metal-oxide nanostructures.

This paper compares the sensitivity between JL-DM-DG-MOSFET with and without gate-stack-based biosensors. The threshold voltage and I_on_/I_off_ ratio are compared between both the devices. The analog/RF characteristics are investigated for JL-DM-DG-MOSFET with and without gate-stack-based biosensors.

This paper is arranged as follows: Section 2 describes the biosensors’ structure with simulation models. The working principle of the biosensors is described in Section 3. Section 4 describes the sensitivity, linearity, and analog/RF characteristics. Section 5 concludes the paper.

## 2. Device Structure and Simulation Framework

**Figure 1:**
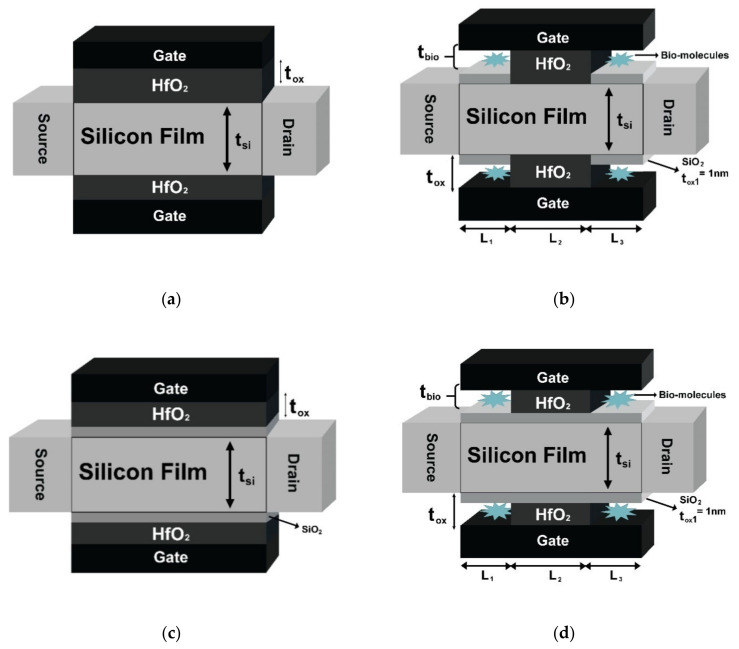
Device structure of (**a**) n-type junctionless DM-DG-MOSFET; (**b**) n-type junctionless DG-MOSFET-based biosensor with cavity; (**c**) n-type junctionless DM-GSDG-MOSFET; (**d**) n-type junctionless DM-GSDG-MOSFET-based biosensor with cavity with channel length of 50 nm.

In this work, different types of DG MOSFET-based biosensors are designed and shown in Figure 1. The n-type junctionless dielectric modulated DG-MOSFET is designed, and the cavity is formed to create a biosensor. The design parameters of biosensors are shown in Table 1. The nanogap cavity length towards the source and drain ends are L_1_ (L_bio_) and L_3_ (L_bio_), both of 10 nm. The gate oxide (HfO_2_) length, L_2_ (L_ox_), is 30 nm. The thickness of the cavity (t_bio_) is 9 nm. The channel (t_si_) and gate oxide (t_ox_) thickness is 10 nm, respectively. The cavity region is considered a native oxide (SiO_2_) layer having a thickness (t_ox1_) of 1 nm.

**Figure 2:**
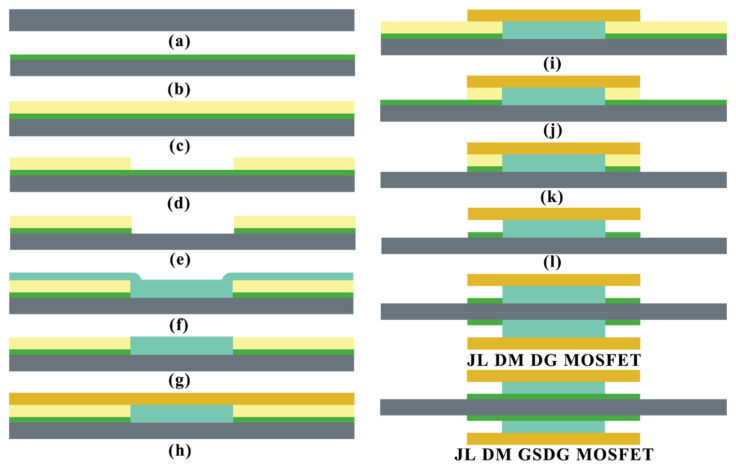
The process flow of the n-type JL-DM-DG-MOSFET and JL-DM-GSDG-MOSFET (**a**–**l**).

In JL-DM-GSDG-MOSFET, a stack of two oxide layers is used in the gate region. The thickness of the SiO_2_ layer (t_ox1_) and the HfO_2_ (t_ox_ − t_ox1_) layer is 1 nm and 9 nm, respectively. The JL-DM-DG-MOSFET-based biosensor with nanogap cavity can be realized using standard IC fabrication [9,10,21,23,24,26,27] and MEMS technology [29]. Figure 2 shows the process flow (a–l) of the DG-MOSFET-based biosensor. The steps followed are given below:(a) firstly, the authors considered an n-type (100) silicon wafer, which is prepared by applying ion implantation, and the silicon layer of 10 nm is formed using thermal oxidation and etching [21,26]; the next step, (b), is the formation of the SiO_2_ layer at the silicon surface by thermal oxidation. (c) Further, using the RF magnetron sputtering technique, the ZnO layer is deposited as a sacrificial layer [29] at the surface of silicon dioxide; in the next step, (d), the ZnO layer of 30 nm at the channel region is etched by using the 1% HCL etchant [29]. (e) Then, SiO_2_ is etched by diluted HF; further, (f) the HfO_2_ layer is deposited at the centre cavity by atomic layer deposition (ALD), and then (g) the unwanted HfO_2_ is removed by using chemical mechanical planarization (CMP) [29]; in the next step, (h), the gate contact is formed by thermal evaporation; after that, etching the (i) gate layer, (j) ZnO layer, and (k) SiO_2_ layer are performed one-by-one at the source and drain side [10]; the last step, (l), is to create a nanogap cavity for the immobilized biomolecules by removing the sacrificial layer. Finally, the n-type JL-DM-DG-MOSFET is prepared. To obtain the n-type JL-DM-GSDG-MOSFET, all steps were followed except step (e).

**Figure 3:**
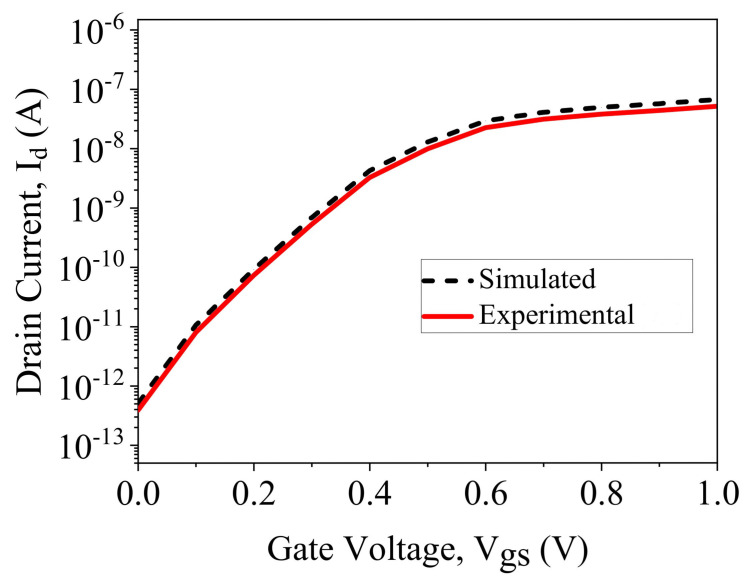
Comparison of the simulated result with the experimental results [30] for n-type JL-DM-GSDG-MOSFET at V_ds_ = 50 mV.

**Figure 4:**
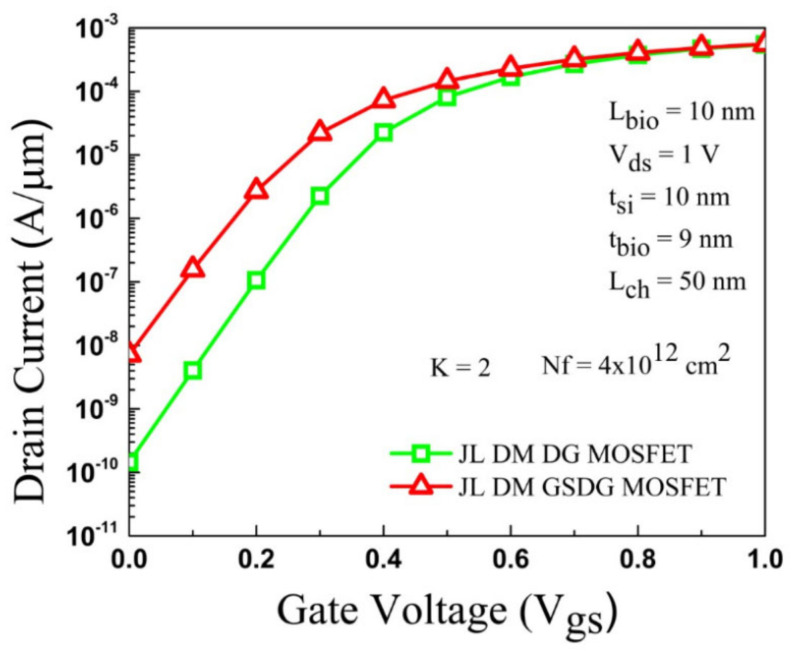
The I_D_-V_gs_ plot of JL-DM-DG-MOSFET and JL-DM-GSDG-MOSFET for L_g_ = 50 nm at V_ds_ = 1V.

Simulation results are calibrated with existing work [30], as shown in Figure 3. The silvaco ATLAS simulator [31] was used to simulate the presented biosensors. The device is simulated with the neutral and charged biomolecule at the nanogap cavity region. The cavity height (t_bio_) depends on the biomolecule used in the nanogap, as shown in Table 2. The presence of neutral biomolecules is simulated by considering t_bio_ = 9 nm, and the dielectric constant varies as K = 2, 3, 4, 5, 6, 7, 8, 9, and 10. The presence of charged biomolecules is considered as an interface fixed charge of N_f_ = ±4 × 10^12^ cm^−2^. The I_D_-Vgs comparison of DG-MOSFET and GSDG-MOSFET is given in Figure 4. From the graph, it can be seen that the DG-MOSFET structure shows a better current ratio. Table 3 displays the comparison of different parameters between both MOSFETs. A lower threshold voltage is observed in the presented structure.

## 3. Working Principle of the Device

The JL-DG-MOSFET [1,2,3,4,5] and JL-GSDG-MOSFET [1,6,7] are considered to design the biosensors. The biomolecule sensing regions are created at the drain and source side as a nanogap cavity. The dielectric constant is changed when the nanogap cavity is filled with the biomolecules. So, the gate capacitance of the cavity region changes due to the changes in the dielectric constant. The electrical characteristics (such as drain current and threshold voltage) are changed due to the change in the capacitance of the cavity regions. The biomolecules used in the simulation are changed due to the changes in dielectric constant K in the cavity. For JL-DM-DG-MOSFET, the gate capacitance per unit area is *C_i_*, where *i* = 1, 2, 3 for region 1 (L_1_), 2 (L_2_), and 3 (L_3_).

$$C_{1}=C_{3}=C_{eff}$$

$$C_{eff}=\frac{\varepsilon_{bio}\varepsilon_{ox1}}{\varepsilon_{bio}t_{ox1}+\varepsilon_{ox1}t_{bio}}$$

where $\varepsilon_{ox1}$ is the dielectric constant of SiO_2_.

$$C_{2}=C_{ox}$$

$$C_{ox}=\frac{\varepsilon_{ox}}{t_{ox}}$$

where the dielectric constant of the gate oxide is denoted by ε_ox_ = ε_2_.

For JL-DM-GSDG-MOSFET,

$$C_{1}=C_{3}=C_{eff}$$

$$C_{2}=\frac{\varepsilon_{HfO_{2}}\varepsilon_{ox1}}{\varepsilon_{HfO_{2}}t_{ox1}+\varepsilon_{ox1}t_{HfO_{2}}}$$

where $t_{HfO_{2}}=\left(t_{ox}-t_{ox1}\right)$ and $\varepsilon_{HfO_{2}}=\varepsilon_{2}$.

## 4. Discussion of Simulation Results

### 4.1. Electric Field

**Figure 5:**
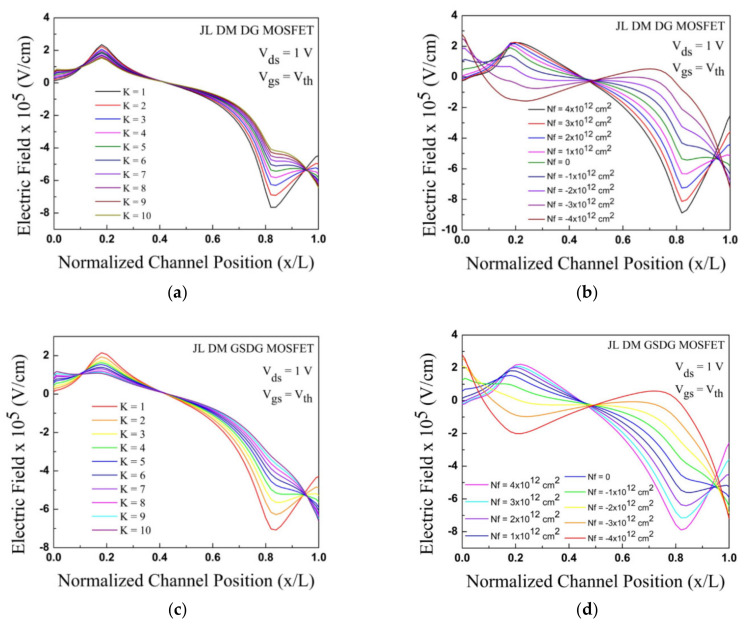
Variation in electric field along the channel: (**a**) effect of K on JL-DM-DG-MOSFET; (**b**) effect of charged biomolecules on JL-DM-DG-MOSFET; (**c**) effect of K on JL-DM-GSDG-MOSFET; (**d**) effect of charged biomolecules on JL-DM-GSDG-MOSFET.

Figure 5 demonstrates the electric field distribution in the channel. The electric field is plotted for dielectric constant of K =1 to K = 10 along the device channel length for both the presented biosensors. The peak electric field is observed for a dielectric constant value of K = 1 at the source cavity, and the lowest value is observed for K = 1 at the drain cavity. Similarly, the electric fields are also plotted for various biomolecules having interface charges of ± 1,  ± 2,  ± 3, and  ± 4 cm^2^ along the device channel length for both the presented biosensors. The electric field towards the drain side is reduced for both types of biomolecules. Deformation of the electric field distribution is observed in the cavity regions.

### 4.2. Surface Potential

**Figure 6:**
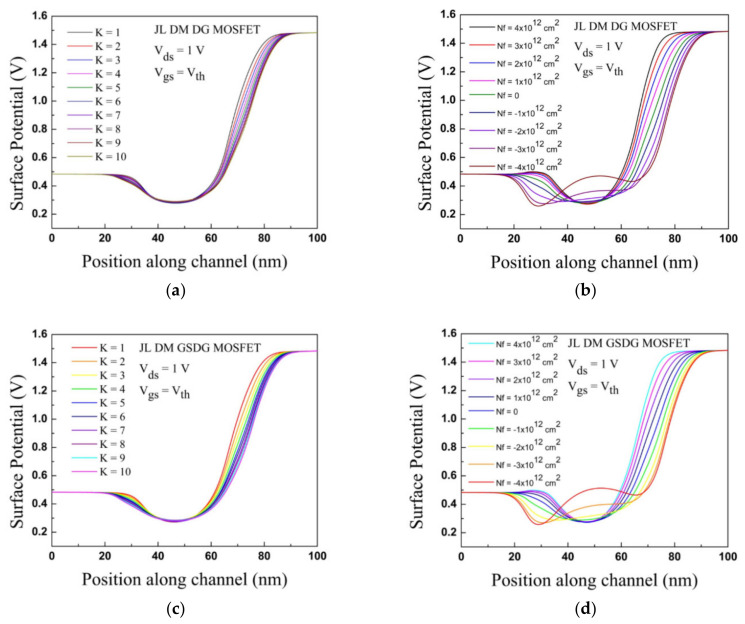
Variation in surface potential along the channel; (**a**) effect of K on JL-DM-DG-MOSFET; (**b**) effect of charged biomolecules on JL-DM-DG-MOSFET; (**c**) effect of K on JL-DM-GSDG-MOSFET; (**d**) effect of charged biomolecules on JL-DM-GSDG-MOSFET.

The surface potential distribution is displayed in Figure 6a,b for the DG-MOSFET. The deformation of the surface potential is observed underneath the cavity regions, and no deformation appears [13] when cavities are not made. The different change in surface potential at the source–channel junction and drain–channel junction is observed due to a linear increase in potential from zero volt at the source electrode and applied drain voltage at the drain terminal. The surface potential at the source–channel junction is less, and at the drain–channel junction, it is more as shown in Figure 6. Therefore, when the cavities are occupied by air, the surface potential is increased by 200 mV at the source–channel junction, and increased by 1200 mV at the drain–channel junction.

The potential profile varies with the dielectric constant for neutral biomolecules present in the cavities. When K varies from 1 to 10, the potential is decreased by 20 mV at the source side and 30 mV at the drain side. For charged biomolecules having magnitude of 4 × 10^12^ cm^2^, the potential is increased by 2 mV due to positively charged biomolecules and decreased by 220 mV due to negatively charged biomolecules at the source–channel junction. At the drain–channel junction, the potential is decreased by 700 mV for negatively charged biomolecules (4 × 10^12^ cm^2^). Under the gate oxide, the potential is increased by 470 mV for the negatively charged biomolecules at the threshold voltage. Here, K = 5 is considered for charged biomolecules to compute the surface potential distribution.

For GSDG-MOSFET, the surface potential profile is displayed in Figure 6c,d. The potential is decreased by 210 mV and increased by 1210 mV at the source and drain side, respectively, when cavities are occupied by air. The potential profile in cavity regions depends on K. For the positively charged biomolecules, the potential is increased by nearly 2 mV at the source side and decreased by 230 mV for the negatively charged biomolecules. When charged biomolecules change from positive to negative at the drain side, the potential decreases by 700 mV.

### 4.3. Energy Band Diagram

**Figure 7:**
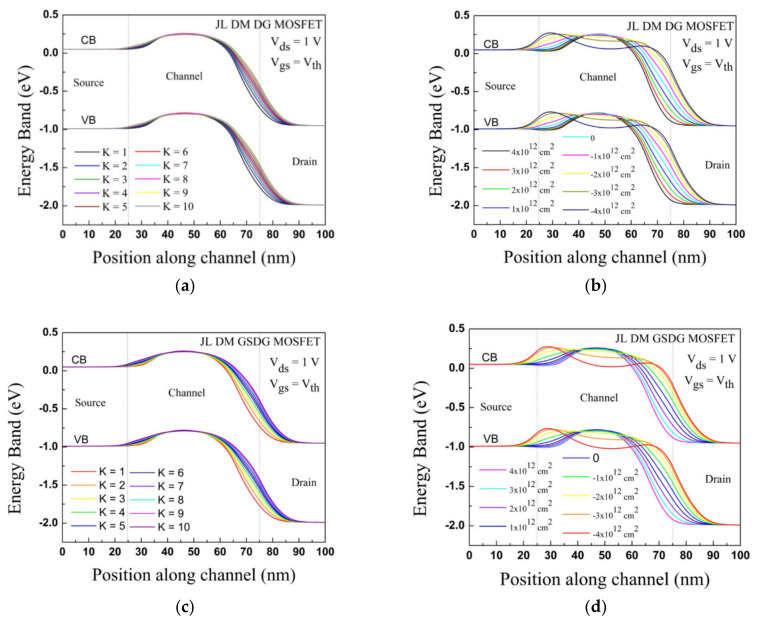
Plot of the energy band diagram for JL-DM-DG-MOSFET and JL-DM-GSDG-MOSFET at V_gs_ = V_th_: (**a**,**c**) neutral biomolecules; (**b**,**d**) charged biomolecules.

In Figure 7, the energy band diagram is displayed after forming cavities at gate oxide for both the devices considering neutral and charged biomolecules at V_ds_ = 1V and V_gs_ = V_th_. The energy band profiles are downward towards the drain region for the junctionless devices, so the carrier injection from source to drain is easy.

Figure 7a,c show the deformation in the EB profile, which is underneath the cavities at the source and drain ends due to neutral biomolecules. Figure 7b,d illustrate the EB profile in the presence of charged biomolecules, where deformation is observed towards the source and drain side. In the source side under the cavity, the EB profile increases (decreases) due to negative (positive) charged biomolecules at CB and VB compared to the drain region.

### 4.4. Drain Current

**Figure 8:**
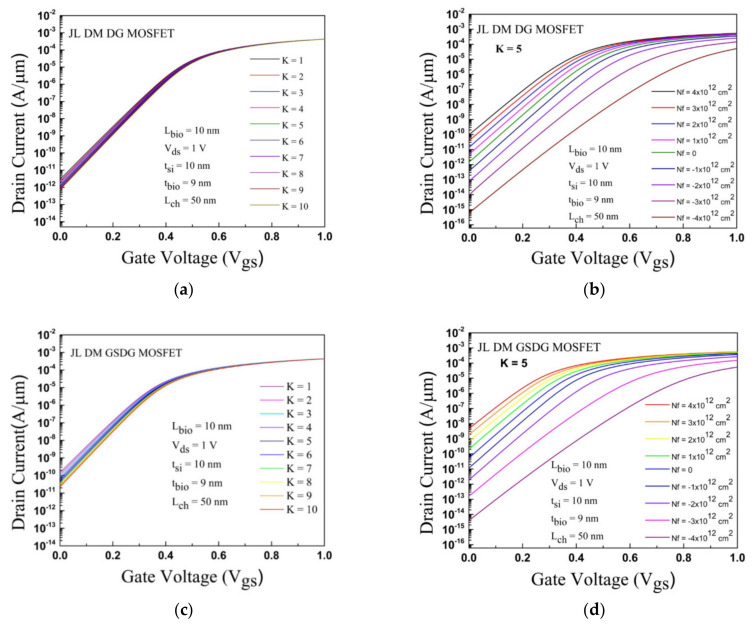
Plot of the transfer characteristics with V_gs_ for JL-DM-DG-MOSFET and JL-DM-GSDG-MOSFET: (**a**,**c**) neutral biomolecules; (**b**,**d**) charged biomolecules.

The transfer characteristic is displayed in Figure 8 for both devices. For neutral biomolecules, the variation of K in the cavities is impacted by the transfer curve of both devices. The OFF-state current (I_off_) exhibits an adequate change from 4.22 × 10^−12^ A/µm to 1.05 × 10^−12^ A/µm, and the ON-state current (I_on_) has a minor change from 4.28 × 10^−4^ A/µm to 4.21 × 10^−4^ A/µm in the DG-MOSFET as given in Figure 8a. In GSDG-MOSFET, the OFF-state current (I_off_) varies from 1.51 × 10^−10^ A/µm to 2.35 × 10^−11^ A/µm, and the I_on_ varies from 4.42 × 10^−4^ A/µm to 4.30 × 10^−04^ A/µm as shown in Figure 8c.

Therefore, the I_off_ and I_on_ decrease by increasing the dielectric constant. In Figure 8b,d, the plot of transfer characteristics is displayed for both devices with charged biomolecules at K = 5. For positively charged biomolecules, the I_off_ and I_on_ increase in both devices. The I_off_ and I_on_ are decreased for negatively charged biomolecules at K = 5.

### 4.5. Threshold Voltage

**Figure 9:**
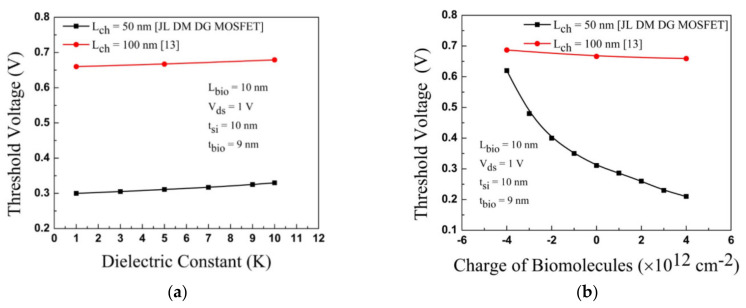
Variation of threshold voltage for JL-DM-DG-MOSFET and [13]: (**a**) neutral biomolecules; (**b**) charged biomolecules.

The threshold voltage is a sensing parameter for biosensors. In Figure 9, the plot of the threshold voltage for the DG-MOSFET is displayed for both biomolecules. The dielectric constant varies from 2 to 10 for neutral biomolecules, and an increase in threshold voltage is detected. The threshold voltage is increased by increasing the negatively charged biomolecules in the cavities. A decrement in the threshold voltage is found with increasing the positively charged biomolecules.

**Figure 10:**
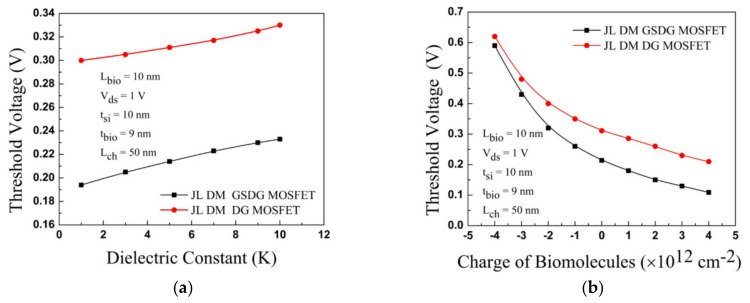
Variation of threshold voltage for JL-DM-DG-MOSFET and JL-DM-GSDG-MOSFET: (**a**) neutral biomolecules; (**b**) charged biomolecules.

Figure 10a compares the threshold voltage between DG-MOSFET and GSDG-MOSFET for biomolecules (neutral and charged). When K varies from 5 to 10, the threshold voltage has a minor difference (20 mV) for GSDG-MOSFET. For DG-MOSFET, a considerable change in threshold voltage (39 mV) is observed by changing K from 1 to 10. The threshold voltage is varied-by 105 mV and 376 mV due to the variation of positive and negative charged biomolecules, respectively, in GSDG-MOSFET at K = 5, given in Figure 10b. The threshold voltage is varied by 101 mV and 309 mV in DG-MOSFET due to the variation of positive and negative charged biomolecules.

### 4.6. Sensitivity

The sensitivity of the biosensor having neutral and charged biomolecules, respectively, is given as

(1) $$\Delta =\left(K=1\right)-V_{th}\left(K>1\right)$$

(2) $$\Delta =\left(Neutral\;Biomolecule\right)-V_{th}\left(Charged\;Biomolecule\right)$$

**Figure 11:**
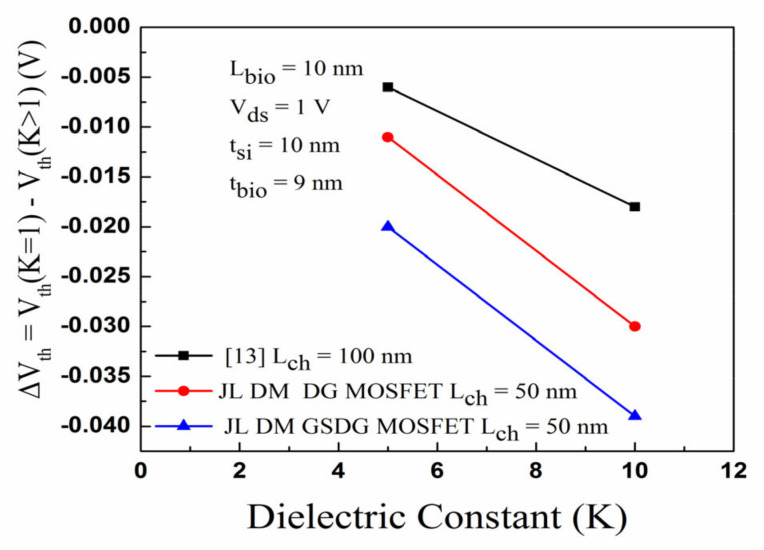
Plot of the sensitivity factor (ΔV_th_ ) for JL-DM-DG-MOSFET, JL-DM-GSDG-MOSFET, and [13] for neutral biomolecules.

**Figure 12:**
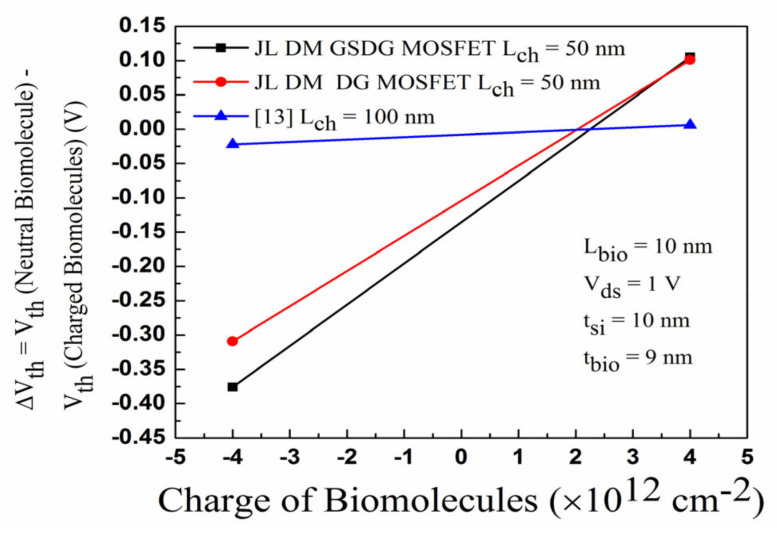
Plot of the sensitivity factor (ΔV_th_) for JL-DM-DG-MOSFET and JL-DM-GSDG-MOSFET, for charged biomolecules.

Figure 11 shows a comparative sensitivity assessment for the DG-MOSFET with [13]. K varies from 1 to 10, and the sensitivity factor is changed by 30 mV (18 mV) in the DG-MOSFET [13]. The ΔV_th_ changes by 101 mV and 309 mV, respectively, for positively charged and negatively charged biomolecules. Figure 11 shows that DG-MOSFET has a better sensitivity factor than [13]. Figure 12 displays the sensitivity of the DG-MOSFET and GSDG-MOSFET for charged biomolecules. The sensitivity factor is changed by 105 mV/376 mV due to the positively/negatively charged biomolecules. The I_on_/I_off_ ratio is evaluated for all the devices.

**Figure 13:**
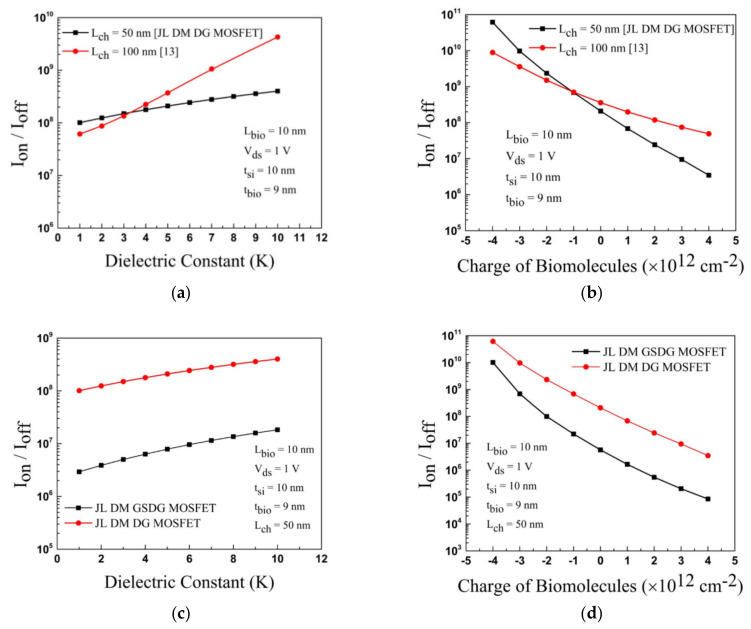
Plot of the I_on_/I_off_ ratio for JL-DM-GSDG-MOSFET and [13]: (**a**) neutral biomolecules; (**b**) charged biomolecules. Plot of the I_on_/I_off_ ratio for the JL-DM-GSDG-MOSFET and JL-DM-GSDG-MOSFET: (**c**) neutral biomolecules; (**d**) for charged biomolecules.

**Figure 14:**
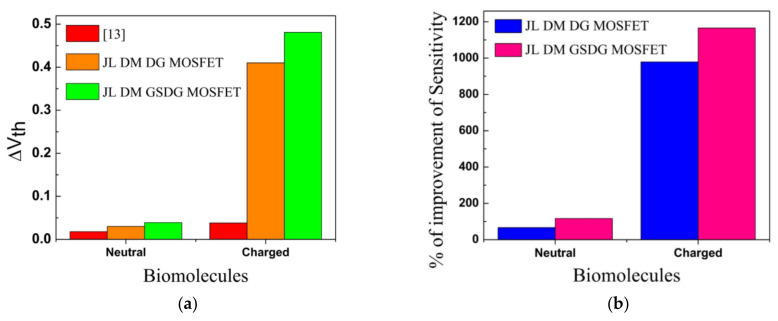
(**a**) Threshold voltage sensitivity of JL-DM-DG-MOSFET, JL-DM-GSDG-MOSFET, and reference [13]; (**b**) sensitivity improvement in JL-DM-DG-MOSFET and JL-DM-GSDG-MOSFET over [13].

In Figure 13a, the plot of I_on_/I_off_ ratio versus the dielectric constant is displayed for neutral biomolecules. The I_on_/I_off_ ratio is increased in both devices. In Figure 13b, the I_on_/I_off_ ratio increases due to negatively charged biomolecules and decreases due to positively charged biomolecules for both devices. Figure 13c,d show the I_on_/I_off_ ratio plot. The sensitivity of DG-MOSFET and GSDG-MOSFET-based biosensors is compared with [13]. The sensitivity is higher in the GSDG-MOSFET-based biosensor followed by DG-MOSFET and [13], as shown in Figure 14a. Sensitivity is improved by 116.66% (66.66%) for the GSDG-MOSFET (DG-MOSFET)-based biosensor compared with [13] for neutral biomolecules. The sensitivity improvement for charged biomolecules is 1165.78% (978.94%) for GSDG-MOSFET (DG-MOSFET)-based biosensors compared to [13] as shown in Figure 14b. Table 4 shows the sensitivity of a JL-DM-GSDG-MOSFET biosensor compared with available JL-MOSFET-based biosensors. The JL-DM-GSDG-MOSFET biosensor shows better sensitivity than the others in the presence of charged biomolecules.

### 4.7. Analog/RF Performance

**Figure 15:**
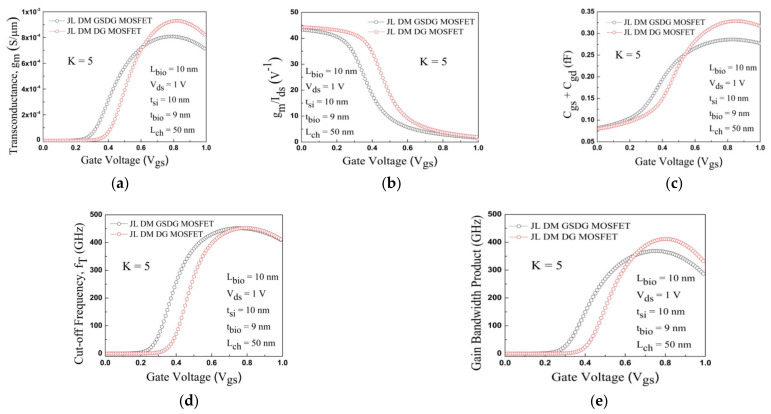
Plot of (**a**) g_m_, (**b**) g_m_/I_ds_, (**c**) C_gs_ + C_ds_, (**d**) f_T_, and (**e**) GBP with gate voltage for JL-DM-DG-MOSFET and JL-DM-GSDG-MOSFET biosensor at K = 5.

The efficiency of the biosensors was investigated in terms of g_m_, g_m_/I_ds_, C_gg_, f_T_, and GBP. For analog/RF performance, a higher g_m_ is required for superior carrier transport efficiency. The plot of the transconductance g_m_ with gate voltage is displayed in Figure 15a, where g_m_ falls at a higher gate voltage for both the devices. The higher value of g_m_/I_ds_ indicates the low power dissipation at the capacitive load circuits. The plot of g_m_/I_ds_ versus gate voltage is displayed in Figure 15b. Figure 15c indicates the plot of C_gs_ + C_gd_ versus the gate voltage. The C_gg_ is less in the subthreshold region and increases gradually at higher gate voltage due to the low inversion charge. The plot of the cut-off frequency ($f_{T}=g_{m}/2\pi C_{gg}$) with V_gs_ is displayed in Figure 15d. Both the devices have a high f_T_ at a high gate voltage due to the total gate capacitance and transconductance. Figure 15e displays the plot of the gain-bandwidth product ($GBP=g_{m}/20\pi C_{gd}$) with gate voltage. The GBP falls at the higher gate voltage for both devices. Hence, both biosensor devices may be used for low-power applications.

### 4.8. Noise Characteristic

**Figure 16:**
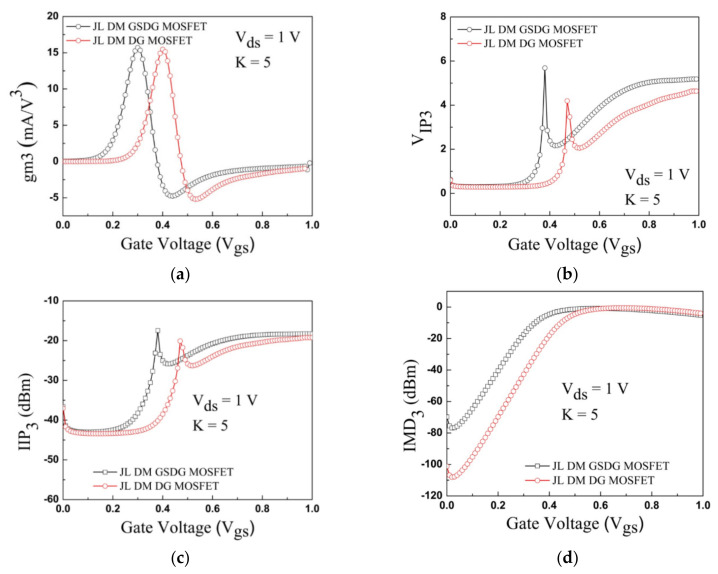
Variation in (**a**) *gm3*, (**b**) V_IP3_, (**c**) IIP_3_, and (**d**) IMD_3_ with gate voltage for JL-DM-DG-MOSFET and JL-DM-GSDG-MOSFET biosensor at K = 5.

The nonlinearity and distortion of the biosensor degrade the sensing capability and signal-to-noise performance [8]. Here, the noise characteristics of the DG-MOSFET and GSDG-MOSFET-based biosensors are compared considering the same device dimensions. Figure 16a displays the plot of the gm3 with gate voltage. The peak of gm3 shows the nonlinearity of the biosensor, which should be low [12]. The peak of the gm3 is almost the same for both biosensors. The signal-to-noise performance is improved by considering the DC bias point close to the zero crossover point. The plot of the V_IP3_ with gate voltage is displayed in Figure 16b. The peak value of the V_IP3_ is high in GSDG-MOSFET at a lower gate voltage than the DG-MOSFET, which indicates that the GSDG-MOSFET is a less noise-affected biosensor [8,9,10,11,12]. The V_IP3_ peak for the GSDG-MOSFET design reflects the cancellation of the third-order nonlinearity coefficient by the device and the internal feedback around the second-order nonlinearity; this reduces the effect of noise. In Figure 16c, the plot of the IIP_3_ with V_gs_ is displayed. The higher value of IIP_3_ indicates less distortion for better sensitivity of the devices [8,9,10,11,12]. The GSDG-MOSFET achieves a higher value of IIP_3_ than the DG-MOSFET at a lower gate voltage. The plot of the IMD_3_ with V_gs_ is displayed in Figure 16d. The value of IMD_3_ is less in GSDG-MOSFET than the DG-MOSFET, indicating that the GSDG-MOSFET performs better in noisy environments [8,9,10,11,12].

## 5. Conclusions

This work reports the design and sensitivity analysis of the n-type JL DM GSDG MOSFET and n-type JL DM DG MOSFET-based biosensors for neutral and charged biomolecules. The sensitivity of both the proposed devices is compared, and the sensing performance of the n-type JL DM GSDG MOSFET-based biosensor is better for both neutral and charged biomolecules. The sensitivity improved in JL DM GSDG MOSFET/JL DM DG MOSFET for neutral and charged biomolecules is 116.66%/66.66% and 1165.78%/978.94%, respectively, compared to reference data. The I_on_/I_off_ ratio for the charged biomolecules is higher than the neutral biomolecules. The I_on_/I_off_ ratio for JL DM GSDG MOSFET is nearly 50 times higher than JL DM DG MOSFET-based biosensors. The variation of electric field, surface potential, and energy band diagram for n-type the JL DM GSDG MOSFET and the n-type JL DM DG MOSFET-based biosensor was also carried out with the dielectric constant and equivalent interface charge of bio-molecules. The impact of dielectric constant and equivalent interface charge of biomolecules is also studied. The n-type JL DM GSDG MOSFET-based biosensor has a lower threshold voltage, and the n-type JL DM DG MOSFET-based biosensor shows a higher current ratio. The JL DM GSDG MOSFET is less sensitive to noise than JL DM DG MOSFET, and both biosensors are better for low-power applications. Thus, the proposed n-type JL DM GSDG MOSFET can be considered a better biosensor than the n-type JL DM DG MOSFET.

## Figures and Tables

**Table 1:** Device Design parameters of JL-DM-DG-MOSFET and JL-DM-GSDG-MOSFET.

Symbol	Quantity	Ref. [13]	JL-DM-DG-MOSFET	JL-DM-GSDG-MOSFET
t_si_ (nm)	Silicon thickness	10	10	10
L (nm)	Channel length	100	50	50
V_gs_ (V)	Gate voltage	1	1	1
V_ds_ (V)	Drain source voltage	1	1	1
t_ox1_ (nm)	Thickness of the SiO_2_ in cavity	1	1	1
t_ox1_ (nm)	Thickness of the SiO_2_ at gate dielectric	0	0	1
L_ox_ (nm)	Length of the HfO_2_ Layer	50	30	30
t_ox_ (nm)	Thickness of the HfO_2_ Layer	10	10	9
L_bio_ (nm)	Length of the nanogap cavity	10	10	10
t_bio_ (nm)	Thickness of the nanogap cavity	9	9	9
ϕ _m_ (eV)	Work function	NR	4.8	4.8
N_A_ (cm^-3^)	Channel doping	1 × 10^19^	4.20 × 10^18^	4.20 × 10^18^
ε *2*	Permittivity of the gate oxide	Al_2_O_3_	25	25
N_f_ (cm^-2^)	Interface fixed Charge	±4 × 10^12^	±4 × 10^12^	±4 × 10^12^

**Table 2:** List of biomolecules with their permittivity values.

Biomolecule	t_bio_ (nm)	ε *_bio_*
APTES	0.9 [10,28]	3.57 [10,13,16,25]
Biotin	0.6 [10]	2.63 [10,13,16]
Protein	4–10 [32]	2.50 [13,16]
Streptavidin	6.1 [10]	2.1 [10,13,16]
DNA	6 [23]	1–64 [23]

**Table 3:** Performance comparison between the JL-DM-DG-MOSFET-based biosensor and the JL-DM-GSDG-MOSFET-based biosensor.

Symbol	Quantity	JL-DM-DG-MOSFET	JL-DM-GSDG-MOSFET
SS (mV/dec)	Subthreshold swing	70.32	80.74
*I_off_ (A/µm)*	OFF-state current	1.45 × 10^−10^	1.45 × 10^−9^
*I_on_ (A/µm)*	ON-state current	5.49 × 10^−4^	5.56 × 10^−4^
*V*_th_ (V)	Threshold voltage	0.199	0.086
*I*_on_ */I*_off_	Current ratio I_on_/I_off_	3.80 × 10^6^	7.75 × 10^4^

**Table 4:** Comparison of sensitivity metric of JL-DM-GSDG-MOSFET biosensor with the existing literature.

Device Parameter	SM-DG [13]	JL-GSSRG [33]	Gate Underlap [34]	Split Gate [35]	DM-DG [36]	JL-DM-GSDG [Proposed Work]
Channel length	100nm	50 nm	100nm	225 nm	100 nm	50 nm
Length of Cavity	25 nm	25 nm	25 nm	175 nm	25 nm	10 nm
Thickness of cavity	9 nm	10 nm	9nm	9nm	9 nm	9 nm
gate oxide	Al_2_O_3_	HfO_2_+ SiO_2_	T iO_2_+ SiO_2_	HfO_2_	T iO_2_ + SiO_2_	HfO_2_ + SiO_2_
Channel thickness	10 nm	20 nm	10 nm	10 nm	10 nm	10 nm
Sensitivity (for neutral biomolecules)	0.08 *V*	0.175 *V*	0.2 *V*	0.22 *V*	0.227 *V*	0.04 V
Sensitivity (for charged biomolecules)	0.27 *V*	-	0.165 *V*	0.35 *V*	0.36 *V*	0.481V
Sensitivity I_on_/I_off_ ratio	10^10^	10^9^	10^9^	–	10^13^	10^11^

## Data Availability

Not applicable.
